# Effect of Pre-Oxidation Treatment of Nano-SiC Particulates on Microstructure and Mechanical Properties of SiC/Mg-8Al-1Sn Composites Fabricated by Powder Metallurgy Combined with Hot Extrusion

**DOI:** 10.3390/ma9120964

**Published:** 2016-11-26

**Authors:** Chuan-Peng Li, Zhi-Guo Wang, Min Zha, Cheng Wang, Hong-Chen Yu, Hui-Yuan Wang, Qi-Chuan Jiang

**Affiliations:** 1 State Key Laboratory of Automotive Simulation and Control, Key Laboratory of Automobile Materials of Ministry of Education & School of Materials Science and Engineering, Nanling Campus, Jilin University, No. 5988 Renmin Street, Changchun 130025, China; licp12@mails.jlu.edu.cn (C.-P.L.); wangzg11@mails.jlu.edu.cn (Z.-G.W.); chengwang@jlu.edu.cn (C.W.); hcyu14@mails.jlu.edu.cn (H.-C.Y.); jqc@jlu.edu.cn (Q.-C.J.); 2 Center of Analytical and Testing, Beihua University, No. 3999 Binjiang East Road, Jilin 132013, China

**Keywords:** magnesium, nano-SiC particulates, pre-oxidation parameters, powder metallurgy, tensile properties

## Abstract

Nano-SiC particulates (n-SiC_p_) reinforced Mg-8Al-1Sn (AT81) composites with different pre-oxidation parameters were fabricated by powder metallurgy (P/M) process combined with hot extrusion. The effects of pre-oxidization treatment of n-SiC_p_ on the microstructure and tensile properties of 0.5 vol % n-SiC_p_/AT81 composites were investigated accordingly. The distribution of n-SiC_p_ with different pre-oxidation parameters was homogeneous in the composites. Moreover, it was found that a thin MgAl_2_O_4_ layer formed at the interface when the n-SiC_p_ were pre-oxidized at 1073 K for 2 h, while the MgAl_2_O_4_ layer became much thicker with pre-oxidization temperature increasing to 1273 K for 2 h. After an appropriate pre-oxidization treatment of n-SiC_p_ at 1073 K for 2 h, the as-extruded 0.5 vol % n-SiC_p_/AT81 composites exhibited an enhanced strength. It was found that the yield strength (YS) and ultimate tensile strength (UTS) increased from 168 MPa and 311 MPa to 255 MPa and 393 MPa compared with the as-extruded AT81 alloy, reflecting 51.8% and 26.4% increments, respectively. The improvement of mechanical properties should be mainly attributed to the grain refinement and homogeneous distribution of n-SiC_p_ in the composites. Moreover, a well-bonded interface and the formation of an appropriate amount of interfacial product (MgAl_2_O_4_) benefited the material’s mechanical properties.

## 1. Introduction

Magnesium (Mg) alloys, the lightest known commercial structural alloys, show very attractive potential application in aerospace and automobile industries due to their low stiffness, high elastic modulus and high specific strength [1,2,3,4,5,6]. In recent decades, researchers have become increasingly interested in enhancing the performance of Mg alloys for wider application [7]. Although high strength can be achieved by using cost intensive magnesium alloys, other properties, e.g., a higher elastic modulus and a lower coefficient of thermal expansion, can be realized only by the addition of strong and stiff second phases to magnesium [8]. As well known, adding ceramic particulates to the matrix, i.e., preparing magnesium metal matrix composites (MMCs), is an important route to enhance the strength of Mg alloys. Particulate reinforcements can significantly increase the matrix mechanical strength such as SiC particulates, which have the advantages of high elastic modulus and hardness [9,10,11]. A relatively small amount of nano-SiC particulates can significantly improve the mechanical properties of Al matrix [12]. Matin et al. [13] fabricated the n-SiC_p_/AZ80 magnesium matrix composites by stir casting method, and found that the tensile strength and the ductility values increased with the adding n-SiC_p_ content accompanied by reduction of the grain size. There are also various methods to fabricate MMCs, such as squeeze casting [14], stir casting [15], mechanical alloying [16], powder metallurgy [17,18], etc. Furthermore, the distribution of particulate reinforcements is one of the important factors in MMCs fabrication process. In the P/M process, as well known, particulates that are mixed sufficiently can lead to the uniform distribution in the MMCs, which is helpful to improving mechanical properties of the composites [19].

There is also strong interest in developing a novel alloy system that provides low-cost, high mechanical strength and thermal stability. Recently, the addition of Sn to Mg alloys has also garnered attention as it can improve the mechanical properties at room and elevated temperatures [20,21,22]. It is well accepted that the addition of Al can increase the tensile strength of alloy materials which offer a beneficial combination of room temperature mechanical properties and low cost [23,24,25]. Moreover, one of the important factors in improving mechanical properties is the interface between the SiC_p_ and the matrix in SiC particulates reinforced Mg-based composites. The possible interfacial reactions can be expressed as follows [26,27,28,29,30,31]:

5Mg + 2Al + 2SiO_2_ = MgAl_2_O_4_ + 2Mg_2_Si (1)

4Mg + SiO_2_ = 2MgO + Mg_2_Si (2)

4Al + 3SiO_2_ = 2Al_2_O_3_ + Si (3)

MgO + Al_2_O_3_ = MgAl_2_O_4_ (4)

2MgO + 4Al + 3SiO_2_ = 2MgAl_2_O_4_ + 3Si (5)

The addition of Al can promote the formation of MgAl_2_O_4_ at the interface between the n-SiC_p_ and matrix, meanwhile enhancing the wetting behavior of the nano-SiC particles with the matrix. Li et al. [32] found that the enhancement effect of MgAl_2_O_4_ is better than Al_4_C_3_ in the Al matrix composites. Surface treatment techniques, such as the pre-oxidation of SiC particulates, have also been adopted by several researchers in effort to obtain optimal interfacial reaction products [27,28]. After the pre-oxidation process, SiC_p_ forms a thin layer of SiO_2_ on the surface. The layer of SiO_2_, which prevents direct contact between SiC and the matrix, is believed to form a stable interfacial structure between the SiC and the Al matrix [29]. Under the same fabrication process, the thickness of the SiO_2_ layer influence mechanical properties of the composites. The SiO_2_ layer must have appropriate thickness for the composites to have superior mechanical properties. Most studies on this subject mainly focused on controlling the interface layer through optimizing pre-oxidation conditions such as pre-oxidation temperature and holding time [33]. The effects of interface layer thickness with different pre-oxidation parameters on the microstructures and tensile properties of AT81 composites have yet to be reported.

In this study, the effects of nano-SiC particulates with different pre-oxidation parameters on the microstructures and tensile properties of 0.5 vol % n-SiC_p_/AT81 composites were investigated, considering that the 0.5 vol % n-SiC_p_ addition can lead to superior properties in the n-SiC_p_/AT81 composite according to our previous study. The purpose of the present study was to determine the optimal pre-oxidation parameters of n-SiC_p_, including pre-oxidation temperature, pre-oxidation time and the appropriate thickness of the interface layer for high tensile properties. The results indicate that suitable pre-oxidation conditions of n-SiC_p_ are indeed very important to fabricate n-SiC_p_/AT81 composites with favorable mechanical properties.

## 2. Experimental Section

Commercial powders including magnesium (≥99.9 wt % purity, ~74 μm), aluminum (≥99.8 wt % purity, ~10 μm), tin (≥99.0 wt % purity, ~38 μm) and silicon carbide (≥99.9 wt % purity, ~40 nm) were used as raw materials. The nano-SiC particulates were first pre-oxidized at 973–1273 K for 2 h to form SiO_2_ layers on the reinforcement surfaces. Then elemental powder blends of Mg-8Al-1Sn (wt %) alloy and 0.5 vol % n-SiC_p_/AT81 composites were mixed thoroughly at a rotational velocity of 120 r/min for 12 h in a planetary ball mill (Model: PMQW2L, Chishun, Nanjing, China) and the whole process was conducted under the protection of high purity argon gas. The mass ratio of ball to powder was ~10:1, and 0.5 wt % stearic acid was used as a lubricant. The blends were cold pressed into cylindrical compacts (45 mm in height and 30 mm in diameter) under a pressure of ~70 MPa for 5 min. The cylindrical compacts were heated to 733 K and held at this temperature for 0.5 h in a self-made vacuum hot pressing and sintering furnace (vacuum degree ≤ 0.01 Pa). Then, the cylindrical compacts were subsequently pressed under 105 MPa for 10 min and cooling down to the ambient temperature. The sintered billets were heated to 633 K for 2 h and extruded at 633 K with an extrusion ratio of 12:1 to obtain sheet samples. These extruded plates were then heated to 698 K for 2 h, followed by hot water (about 353 K) quenching. Then, the aging was performed at 448 K for 18 h.

The microstructures of the samples were observed on a scanning electron microscope (SEM, FEI Quanta 200, Hillsboro, OR, USA) equipped with an energy dispersive spectrometer (EDS, Oxford-x-max^n^, London, UK) analyzer, a field emission scanning electron microscope (FESEM, JSM6700F, Tokyo, Japan) and transmission electron microscope (TEM, JEM 2100F, Tokyo, Japan and FEI f20, Hillsboro, OR, USA). Specimens for TEM analysis were thinned via ion milling to about 40 μm (Gatan 691, Pleasanton, CA, USA). Samples for microstructure observations were polished and then chemically etched in an acetic picral solution (20 mL ethanol, 3 mL acetic acid, 1 mL distilled water and 2 g picric acid). The phase constituents were examined by X-ray diffraction (XRD, Model X’Pert PRO PANalytical, Almelo, The Netherlands) with Cu Kα radiation at the voltage of 40 kV with a scanning speed of 0.06°/s. At room temperature, the dog-bone-shaped tensile samples, having a gauge size of 4 mm in width and 30 mm in length, were tested on a servo-hydraulic materials testing system (MTS, MTS810, INSTRON, Boston, MA, USA) at a constant strain rate of 1.0 × 10^−3^ s^−1^. In tensile tests, at least three samples were tested for each condition and the average value was calculated and given.

## 3. Results and Discussion

**Figure 1:**
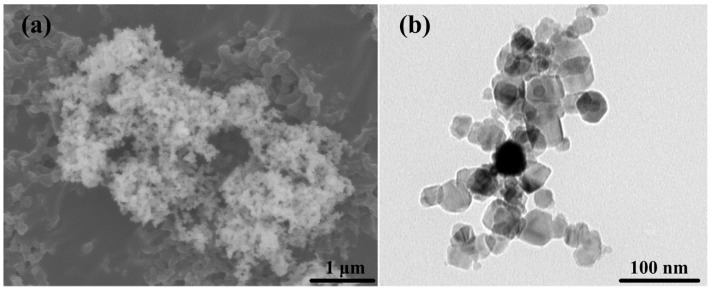
(**a**) FESEM micrographs; and (**b**) TEM image of nano-SiC particles.

Figure 1 shows the FESEM micrographs and TEM images of nano-SiC particles. In Figure 1a, it could be observed that the commercial powders of n-SiC_p_ are highly agglomerated and nearly spherical. As shown in Figure 1b, the initial state of SiC_p_ is mostly equiaxed in shape and about 40 nm in size at high magnification.

**Figure 2:**
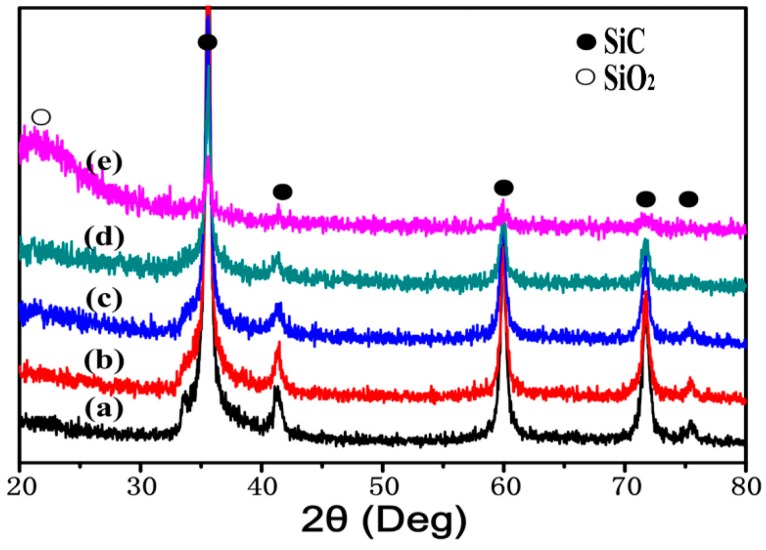
XRD patterns of nano-SiC particulates with different pre-oxidation parameters of: (**a**) untreated; (**b**) 973 K/2 h; (**c**) 1073 K/2 h; (**d**) 1173 K/2 h and (**e**) 1273 K/2 h.

Figure 2 shows XRD patterns of nano-SiC particulates with different pre-oxidation parameters. In Figure 2b–d, it can be seen that SiO_2_ began to appear with increasing temperature compared with the untreated n-SiC_p_ shown in Figure 2a. Finally, steamed bread peak appeared in Figure 2e, which indicated that a mass of SiO_2_ formed on the surface of the nano-SiC particulates as expected.

**Figure 3:**
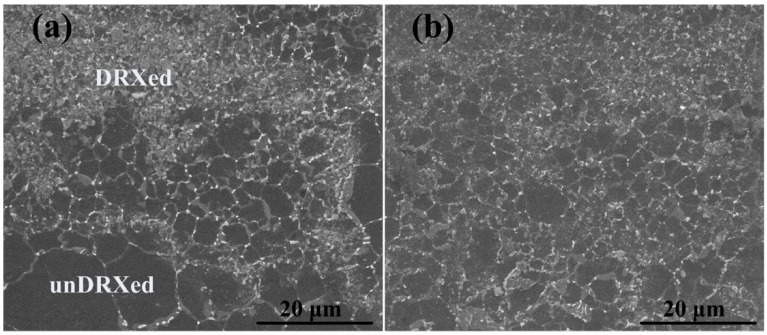
SEM microstructures of the: (**a**) extruded AT81; and (**b**) 0.5 vol % n-SiC_p_/AT81 composites reinforced by 1073 K/2 h treated n-SiC_p_.

Figure 3 shows the SEM microstructures of extruded AT81 and 0.5 vol % n-SiC_p_/AT81 composites reinforced by 1073 K/2 h treated n-SiC_p_. One can see that Mg_17_Al_12_ phases were mainly distributed at grain boundaries. Both quite large and relatively small grains coexisted in the AT81 alloys as shown in Figure 3a. Meanwhile, dynamic recrystallization (DRX) and un-DRXed regions were visible in both materials, while the grain size was more refined in the DRXed regions. However, the 0.5 vol % n-SiC_p_/AT81 composites exhibited the higher fractions of DRXed grain structures as shown in Figure 3b. As compared to the AT81 alloys, the grain size of 0.5 vol % n-SiC_p_/AT81 composites was relatively small due to the combination of recrystallization during hot extrusion and pinning effect resulting from the presence of uniformly distributed n-SiC_p_. As well known, the refinement of grains contributed to the improvement of mechanical properties [34].

**Figure 4:**
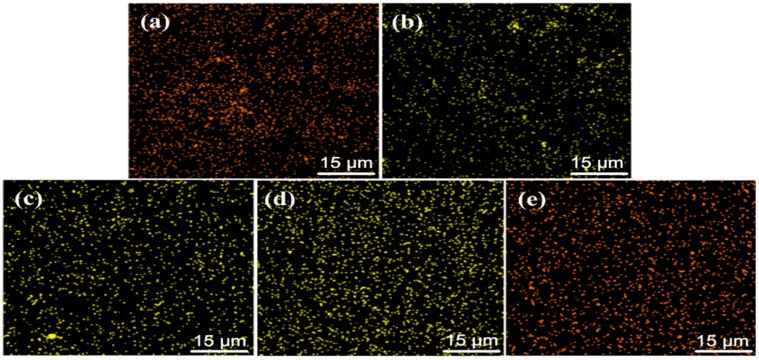
X-ray maps of Si analysis in the extruded 0.5 vol % n-SiC_p_/ AT81 composites reinforced by: (**a**) untreated; (**b**) 973 K/2 h; (**c**) 1073 K/2 h; (**d**) 1173 K/2 h; and (**e**) 1273 K/2 h treated n-SiC_p_.

However, the distribution of n-SiC_p_ in the matrix was difficult to be observed because the average size of SiC particulates was only ~40 nm. To clarify the distribution of n-SiC_p_ in the matrix, typical X-ray maps of Si in the extruded 0.5 vol % n-SiC_p_/AT81 composites reinforced by n-SiC_p_ with different pre-oxidation parameters were shown in Figure 4. In Figure 4, it is revealed that the distribution of Si element was homogeneous, suggesting n-SiC_p_ dispersed homogeneously in the extruded 0.5 vol % n-SiC_p_/AT81 composites. It is worth noting that the homogeneous distribution of particulate is crucial to obtain favorable mechanical properties of the achieved composites.

**Figure 5:**
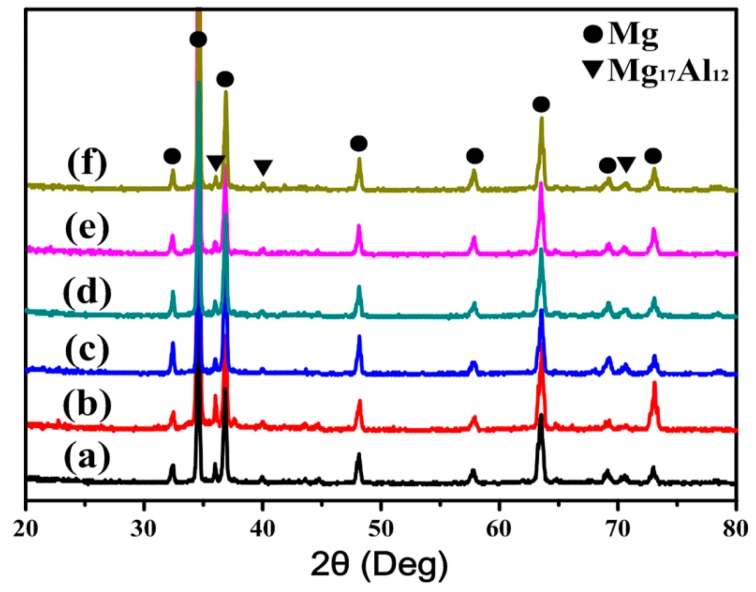
XRD patterns of: (**a**) extruded AT81 and 0.5 vol % n-SiC_p_/AT81 composites reinforced by n-SiC_p_ with different pre-oxidation parameters: (**b**) untreated; (**c**) 973 K/2 h; (**d**) 1073 K/2 h; (**e**) 1173 K/2 h; and (**f**) 1273 K/2 h.

To observe phase constituents, XRD patterns of the extruded AT81 and 0.5 vol % n-SiC_p_/AT81 composites reinforced by n-SiC_p_ with various pre-oxidation parameters are presented in Figure 5. No obvious MgO phase was identified, indicating that the utilization of high purity argon gas and vacuum atmosphere greatly reduced the oxidation of the matrix during fabrication.

**Figure 6:**
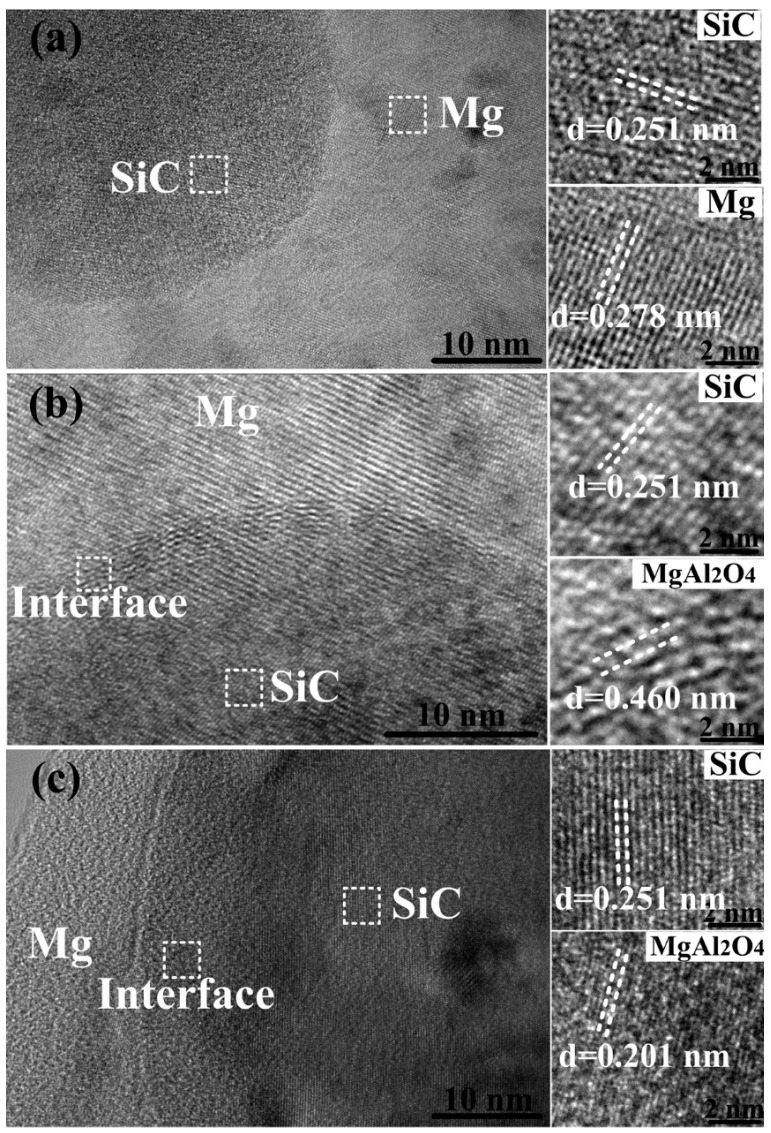
HRTEM images of 0.5 vol % n-SiC_p_/AT81 composites reinforced by: (**a**) untreated; (**b**) 1073 K/2 h; and (**c**) 1273 K/2 h treated n-SiC_p_.

The interface between n-SiC_p_ and the matrix is shown in Figure 6: as observed by HRTEM, showing that the n-SiC_p_ combined well with the matrix. Moreover, based on the interface regions shown in Figure 6a, there was a clean interface with no obvious interfacial products, indicating a good bonding between the n-SiC_p_ and the matrix. The well-bonded interface between n-SiC_p_ and the matrix can benefit the effective transfer of tensile load from the matrix to the hard particulates, thus leading to the improvement of mechanical properties. In the present investigation, it was found that when the n-SiC_p_ was pre-oxidized at 1073 K for 2 h, the MgAl_2_O_4_ formed between n-SiC_p_ and matrix could be discerned, while the interface layer was thin (about 1.0 nm in thickness) as shown in Figure 6b. When pre-oxidation parameter was 1273 K/2 h, MgAl_2_O_4_ was clearly observed and the thickness of interface between the n-SiC_p_ and matrix was about 6–7 nm shown in Figure 6c. In the literature [32,35], Li and Munitz et al. pointed out that the interfacial products produced from Mg, Al, Si and O components. Luo et al. [36] found that pre-oxidation of SiC introduced a thin coating layer of SiO_2_ surface, which was believed to act as an intermediate to form stable interfacial structures. Thereby, MgAl_2_O_4_ layer could form by the reactions among Mg, Al and SiO_2_ layer according the Equations (1)–(5) and became the main reaction product at the SiC/matrix interface [26,27,28,29,30,31,32,35,36]. Moreover, with the increase of pre-oxidation temperature, there would be more SiO_2_ coated on the SiC, meanwhile the reactions among Mg, Al and SiO_2_ layer could be facilitated, accelerating the formation of a thick MgAl_2_O_4_ layer.

**Figure 7:**
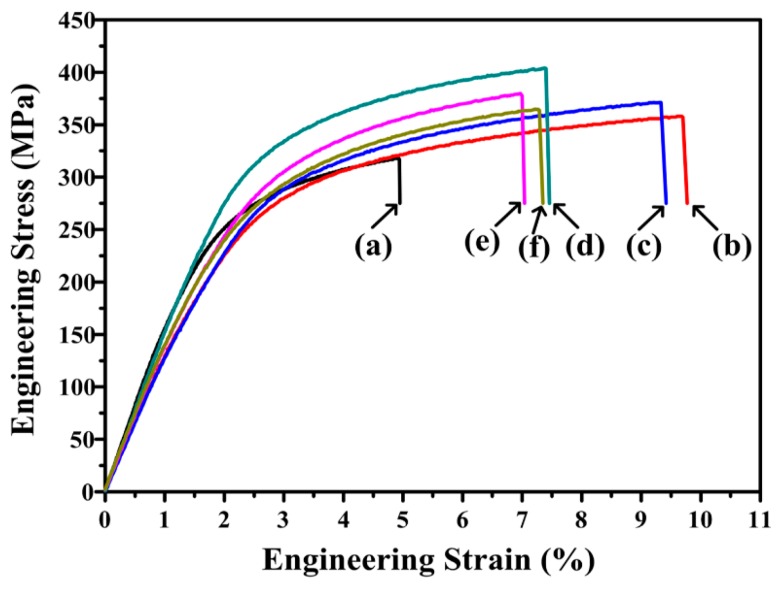
Tensile engineering stress–strain curves of: (**a**) extruded AT81 and 0.5 vol % n-SiC_p_/AT81 composites reinforced by n-SiC_p_ with different pre-oxidation parameters: (**b**) untreated; (**c**) 973 K/2 h, (**d**) 1073 K/2 h; (**e**) 1173 K/2 h; and (**f**) 1273 K/2 h.

The tensile engineering stress–strain curves of extruded AT81 and 0.5 vol % n-SiC_p_/AT81 composites reinforced by n-SiC_p_ with different pre-oxidation parameters are shown in Figure 7. The mean values and standard deviation of the 0.2% offset yield strength (YS), ultimate tensile strength (UTS) and elongation (ε) are summarized in Table 1. As can be seen, the YS, UTS and ε values of the n-SiC_p_ reinforced composites showed significant improvement compared with the unreinforced AT81 alloy at room temperature. The YS and UTS of the extruded AT81 alloy are 168 MPa and 311 MPa, respectively. The YS and UTS of the composite reinforced by untreated n-SiC_p_ are 205 MPa and 364 MPa, respectively, representing 22.0% and 17.0% improvements compared with the particulate-free AT81 alloy. For the 0.5 vol % n-SiC_p_/AT81 composites reinforced by pre-oxidized n-SiC_p_, the tensile strength firstly increased and then decreased with increasing pre-oxidation temperature from 973 to 1273 K at a holding time of 2 h. The composite reinforced by n-SiC_p_ pre-oxidized at 1073 K for 2 h, exhibited the YS and UTS of 255 MPa and 393 MPa, respectively, which was 51.8% and 26.4% higher as compared to the particulate-free AT81 (168 MPa and 311 MPa), 24.4% and 8.0% higher than those of composite reinforced by untreated n-SiC_p_ (205 MPa and 364 MPa), respectively. However, the tensile properties began to decrease as the pre-oxidation temperature further increased to 1273 K, where the YS and UTS dropped to 201 MPa and 362 MPa, respectively. As mentioned earlier (Figure 6), a thin layer (~1 nm in thickness) of MgAl_2_O_4_ was formed at the interface when n-SiC_p_ was pre-oxidized at 1073 K for 2 h, which became much thicker (~6–7 nm in thickness) when pre-oxidized temperature at 1273 K for 2 h. Taken together, it was suggested that the formation of a thin layer MgAl_2_O_4_ was helpful for the bonding strength of interface and could improve the tensile strength of the n-SiC_p_/AT81 composites. However, when the MgAl_2_O_4_ layer exceeded a certain thickness, i.e., about 6–7 nm, the tensile properties tended to decline. Accordingly, the appropriate pre-oxidation parameters for n-SiC_p_ are 1073 K and 2 h to fabricate the 0.5 vol % n-SiC_p_/AT81 composites via P/M process.

**Figure 8:**
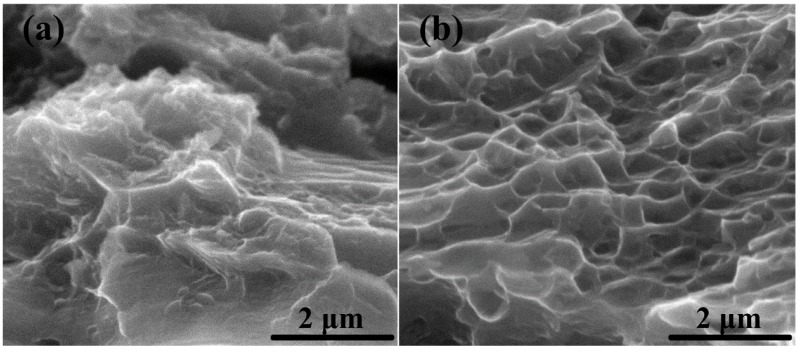
Fracture surfaces of: (**a**) extruded AT81; and (**b**) 0.5 vol % n-SiC_p_/AT81 composites reinforced by pre-oxidized (1073 K/2 h) n-SiC_p_.

Tensile fractographs of extruded AT81 alloy and 0.5 vol % n-SiC_p_/AT81 composites reinforced by n-SiC_p_ with the optimal pre-oxidation parameters (1073 K/2 h) are shown in Figure 8. It can be clearly observed that the fracture of AT81 was brittle, with only a few dimples, as shown in Figure 8a. For 0.5 vol % n-SiC_p_/AT81 composites, numerous dimple-like features, as shown in Figure 8b, were noticed on the fracture surfaces, which may be attributed to the formation of tiny voids in the n-SiC_p_ and composites interfacial areas under tensile deformation and their subsequent coalescence. This indicated that the fracture mode of the 0.5 vol % n-SiC_p_/AT81 composites was ductile and the adhesion of n-SiC_p_/AT81 interfaces was strong, which is beneficial for stress transfer from the matrix to the n-SiC_p_.

## 4. Conclusions

In this study, the 0.5 vol % n-SiC_p_/AT81 composites were successfully fabricated via powder metallurgy combined with hot extrusion. The distribution of n-SiC_p_ with different pre-oxidation parameters was homogeneous in the 0.5 vol % n-SiC_p_/AT81 composites. Moreover, it was found that a thin layer of MgAl_2_O_4_ formed at the interface in the composite when the n-SiC_p_ were pre-oxidized at 1073 K for 2 h, while the MgAl_2_O_4_ layer became much thicker with pre-oxidization temperature increasing to 1273 K for 2 h. Tensile testing revealed that the 0.5 vol % n-SiC_p_/AT81 composite exhibited the highest tensile strength when the pre-oxidation parameters of n-SiC_p_ were 1073 K for 2 h, where the YS and UTS are 255 MPa and 393 MPa, i.e., 51.8% and 26.4% higher than those of the extruded AT81 alloy (168 MPa and 311 MPa), respectively, while ε values kept almost constant. The enhanced tensile properties of the composites were altogether due to the grain refinement, a well-bonded interface and the formation of an appropriate amount of interfacial product (MgAl_2_O_4_) between the n-SiC_p_ and the matrix.

## Figures and Tables

**Table 1:** Tensile properties of the extruded AT81 and 0.5 vol % n-SiC_p_/AT81 composites reinforced by n-SiC_p_ with different pre-oxidation parameters.

Materials	YS/MPa	UTS/MPa	ε/%
AT81	$168_{-18}^{+16}$	$311_{-6}^{+7}$	$4.3_{-0.4}^{+0.2}$
untreated	$205_{-4}^{+7}$	$364_{-6}^{+10}$	$8.8_{-0.8}^{+1.2}$
973 K/2 h	$206_{-26}^{+15}$	$369_{-9}^{+9}$	$8.1_{-1.1}^{+1.7}$
1073 K/2 h	$255_{-18}^{+16}$	$393_{-11}^{+11}$	$5.8_{-0.6}^{+0.6}$
1173 K/2 h	$237_{-9}^{+17}$	$383_{-5}^{+9}$	$6.7_{-1.5}^{+1.9}$
1273 K/2 h	$201_{-8}^{+9}$	$362_{-5}^{+4}$	$6.0_{-0.2}^{+0.2}$
